# Impact of the PPOS protocol on euploidy embryo rates and reproductive outcomes in preimplantation genetic testing cycles: a systematic review

**DOI:** 10.3389/fendo.2025.1595232

**Published:** 2025-09-05

**Authors:** Youwen Mei, Yacong Wang, Li Kuang, Yonghong Lin, Fang Wang

**Affiliations:** Department of Fertility and Sterility, Chengdu Women’s and Children’s Central Hospital, School of Medicine, University of Electronic Science and Technology of China, Chengdu, China

**Keywords:** progesterone-primed ovarian stimulation protocol, preimplantation genetic testing, euploidy embryo rate, reproductive outcomes, controlled ovarian hyperstimulation

## Abstract

The success of assisted reproductive technology (ART) relies on the quality of embryos, particularly the euploid status, which is influenced by controlled ovarian hyperstimulation (COH) protocols. In recent years, the progesterone-primed ovarian stimulation (PPOS) protocol has gained popularity due to its potential benefits. However, the impact of PPOS on euploid embryo rates (EER) and reproductive outcomes remains incompletely understood. Therefore, we conducted this review to comprehensively assess this impact by comparing the PPOS with conventional COH protocols in PGT cycles. The results revealed that the PPOS protocol demonstrated comparable EER and reproductive outcomes to conventional COH protocols in the general population. Among patients with a good prognosis, EER and associated reproductive outcomes with PPOS may be less favorable. However, in individuals with a poor prognosis, PPOS showed comparable or even superior outcomes. Additionally, the timing of cycle initiation, whether in the follicular or luteal phase, had no significant impact on clinical outcomes in patients with diverse ovarian responses.

## 1 Introduction

Controlled ovarian hyperstimulation (COH) is fundamental in assisted reproductive technology (ART), with the primary objective of retrieving multiple oocytes to generate sufficient embryos for transfer. As the field of ART has evolved, the emphasis has gradually shifted from the quantity of embryos to their quality, particularly in achieving euploidy embryos (1). Various studies have reported associations between COH protocols and euploidy embryo rates (EER), presenting a significant challenge for reproductive specialists in determining the optimal COH strategy for patients (2).

The progesterone-primed ovarian stimulation (PPOS) approach, utilizes oral administration of exogenous progesterone to suppress the estradiol-induced luteinizing hormone (LH) surges, offering a viable alternative to traditional COH treatment. This protocol offers several notable advantages by mitigating the risk of ovarian hyperstimulation syndrome, facilitating convenient administration, and providing high cost-effectiveness (3).

In a large retrospective study involving 14,981 recipients of vitrified oocytes from 3,599 donors stimulated with PPOS protocol and 4,998 stimulated with GnRH antagonist protocol, the PPOS group demonstrated comparable clinical outcomes to the GnRH antagonist group in vitrified donor oocyte cycles (4). A meta-analysis encompassing 14 studies with 4,182 participants also revealed that the PPOS protocol improves *in vitro* fertilization (IVF)/intracytoplasmic sperm injection (ICSI) outcomes in women with diminished ovarian reserve (DOR) (5). However, some studies had contradictory results. In a propensity score-matched retrospective cohort study involving 6,520 infertile women aged 20 – 50 years, the GnRH antagonist protocol showed significantly higher cumulative live birth rates (CLBRs) and shorter time to live birth (TTLB) than the progestin-primed ovarian stimulation (PPOS) protocol in unselected IVF patients (6). In a prospective randomized controlled trial at a university-affiliated IVF center, 318 oocyte donors were randomized 1:1 to PPOS or GnRH antagonist protocols. While both groups yielded similar numbers of mature oocytes, retrospective analysis of recipient outcomes showed lower ongoing pregnancy and live birth rates in recipients of PPOS-stimulated donor oocytes (7). Nevertheless, the precise effects of progesterone administration during COH on embryo quality and the underlying mechanisms remain incompletely understood.

Euploidy status is a pivotal aspect in assessing embryo quality and has emerged as a crucial determinant in ART outcomes. As preimplantation genetic testing (PGT) enables the selection of euploid embryos (8), it becomes practical to evaluate the effects of PPOS protocol in PGT cycles. This review aims to comprehensively investigate the impact of PPOS protocol on both the EER and reproductive outcomes in PGT cycles by comparison with other COH protocols. By conducting subgroup analyses, we strive to identify preferences for different patient cohorts, ultimately contributing to the refinement and optimization of ART strategies.

## 2 Methods

### 2.1 Inclusion criteria

**Figure 1:**
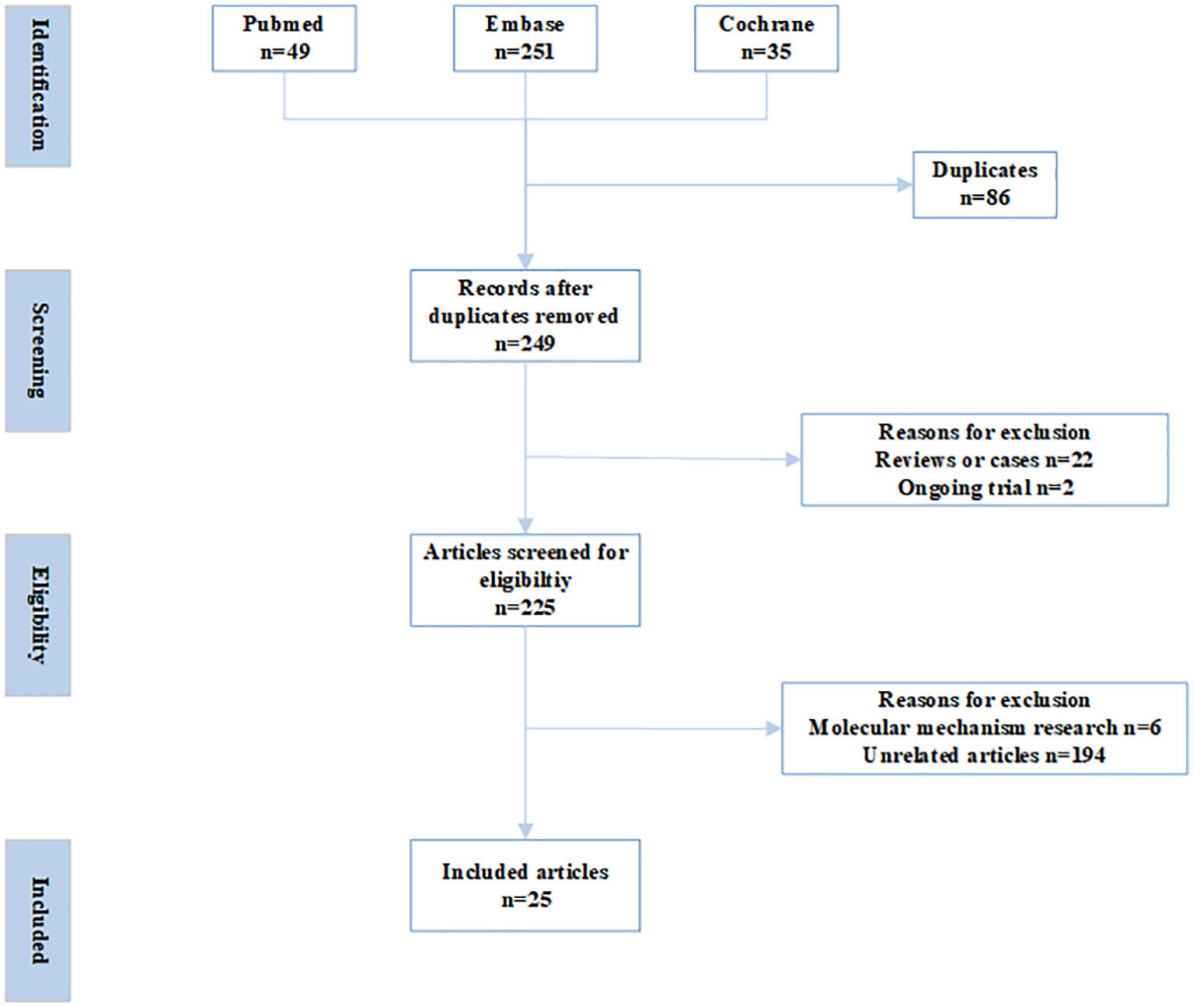
PRISMA flowchart.

Databases of PubMed, Embase, and Cochrane Central Register of Controlled Trials were searched with the following terms: (preimplantation genetic diagnosis OR preimplantation genetic screening OR euploid OR preimplantation genetic test) AND (Controlled ovarian hyperstimulation OR COH OR Ovarian stimulation OR OS OR Controlled ovarian stimulation OR COS OR Ovarian hyperstimulation) AND (Progesterone-Primed Ovarian Stimulation OR PPOS OR progestin-primed ovarian stimulation OR dydrogesterone OR progesterone OR Norethisterone acetate OR Medroxyprogesterone OR MPA) from inception to December, 2024. The inclusion criteria were as follows. Published in English in peer-reviewed journals; irrespective of study-design; studies focusing on the impact of PPOS protocol on euploid embryo rates and reproductive outcomes in PGT cycles. Commentaries, letters, reviews, conference abstracts, and irrelevant studies were excluded (**Figure 1**).

### 2.2 Study selection, data extraction and comprehensive analysis

**Figure 2:**
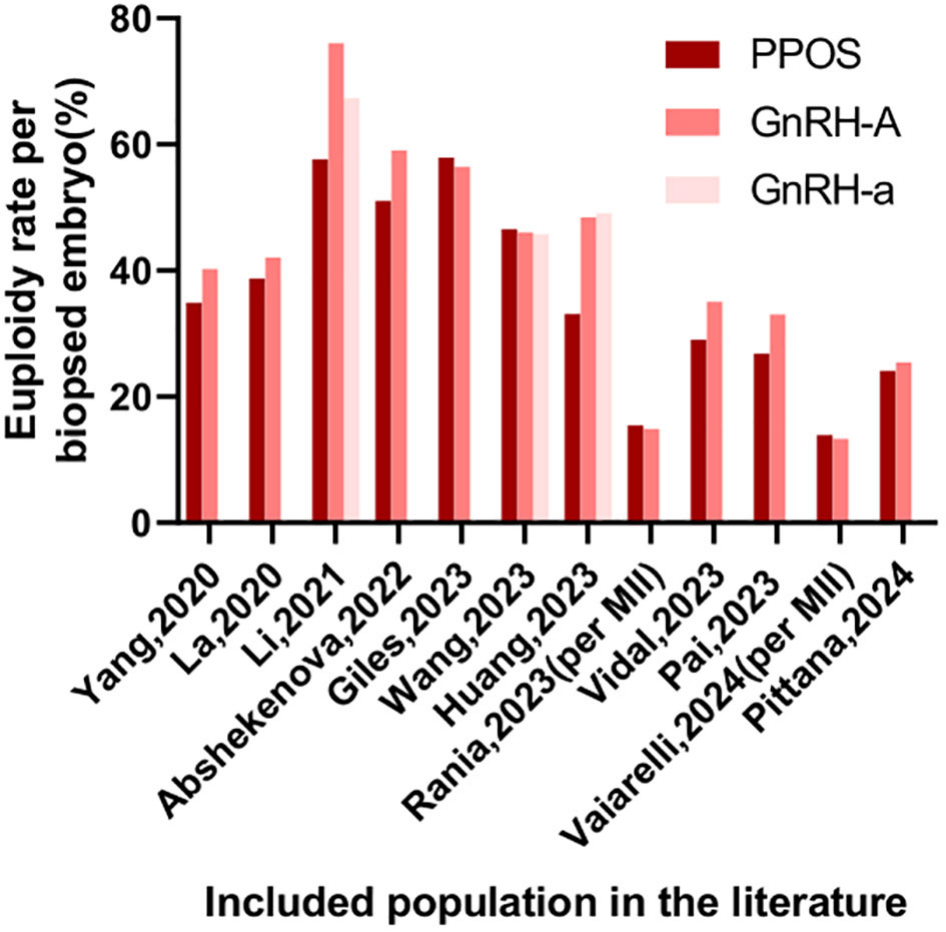
Euploidy Embryo Rate in the included population.

**Figure 3:**
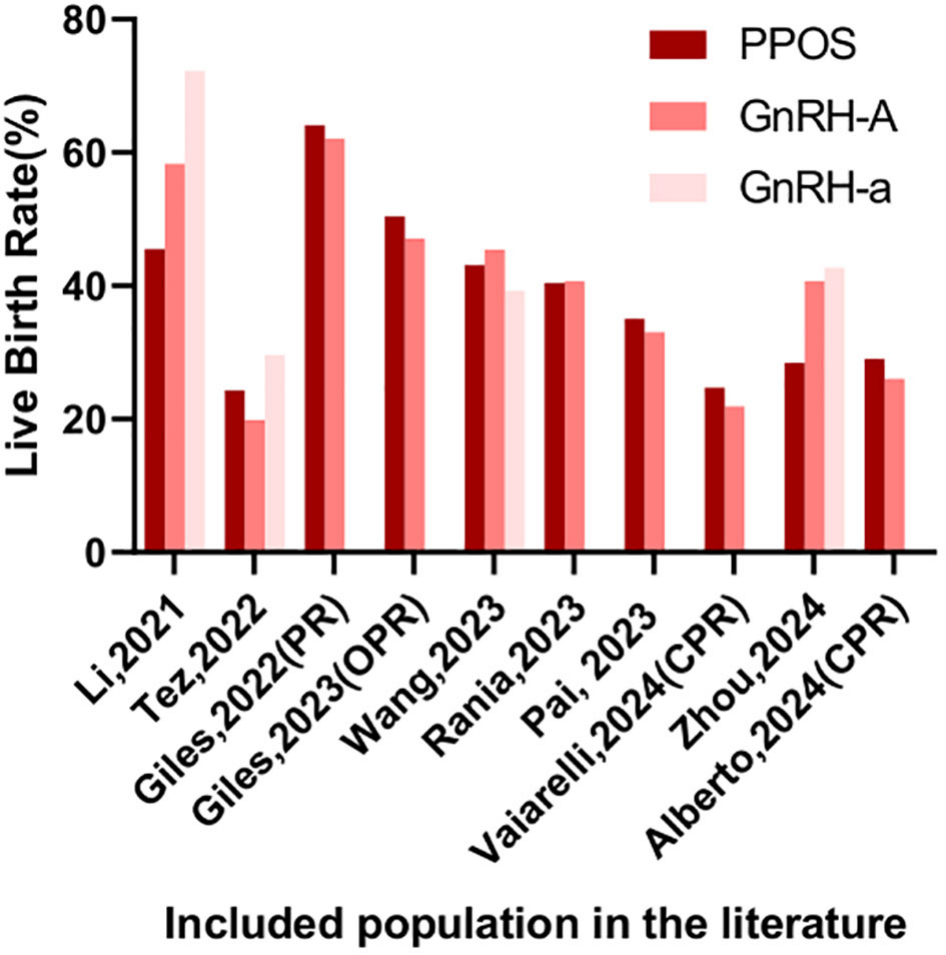
Live Birth Rate in the included population.

Two independent reviewers carried out the initial screening and full-text assessment to determine inclusion. In case of disagreement, consensus was reached through discussion with a third reviewer. The extracted information encompassed details like author names, publication year, study population characteristics, sample size, methodologies, interventions, euploidy embryo rates, and reproductive outcomes, with stratified analyses performed based on demographic features. Due to substantial heterogeneity among the studies, meta-analysis was excluded (**Table 1**, **2**, **Figures 2**, **3**). This review was conducted according to the Preferred Reporting Items for Systematic Reviews and Meta-Analyses guidelines (9). Newcastle-Ottawa Scale (NOS) was used to assess the risk of bias of the included studies.

**Table 1:** The baseline characteristics of the included population.

Reference	Research type	Year	Population	Groups	Subgroups	Sample size	Age	AMH (ng/ml)
Yang, 2020/2022 (18, 19)	Retrospective cohort study	2017-2019	PGT-A	PPOS (DYG)		390		
				GnRH-A		390		
La Marca, 2020 (20)	Prospective case control study	2018-2019	PGT-A (matched)	PPOS (MPA)		48	37 (33 – 39)	3.2(1.9 – 3.9)
				GnRH-A		144	37 (33 – 39)	3.4 (1.8 – 4.1)
Li, 2021 (25)	Retrospective cohort study	2018-2020	PGT-M, <35 years old	PPOS		44		
				GnRH-A		60		
				GnRH-a		43		
Abshekenova, 2022 (22)	Retrospective cohort study	2018-2020	Donor oocyte (same population)	PPOS		123		
				GnRH-A		93		
				long GnRH-a		116		
				GnRH-A		490		
Giles, 2022 (10)	Retrospective cohort study	2017-2021	PGT - A	PPOS		686 c		
				GnRH-A		11775 c		
Giles, 2023 (11)	Retrospective cohort study	2017-2021	PGT - A	PPOS		635		
				GnRH-A		5026		
Huang, 2023 (16)	Retrospective case control study	2019-2022	PGT-A for AMA, RSA, and RIF	PPOS (DYG)		152 c	37.11 ± 4.48	2.27 ± 2.33
				GnRH-A		179 c	34.01 ± 4.88	3.62 ± 2.37
				GnRH-a		126 c	33.82 ± 5.25	3.16 ± 2.08
Rania, 2023 (29)	Prospective case control study	2017-2020	PGT-A for AMA (matched)	PPOS		80		
				GnRH-A		5026		
Wang, 2023 (12)	Retrospective case control study	2019-2021	PGT-A	PPOS		140	38(33 – 41)	2.49(1.12 – 5.59)
				GnRH-A		302	37(33 – 40)	2.7(1.45 – 4.86)
				GnRH-a		146	35(32 – 39)	3.33(2.33 – 4.76)
Zhou, 2023 (26)	Retrospective cohort study	2017-2020	PGT-A, M, SR	PPOS (MPA)	NOR	250	32.65 ± 4.74	3.76 ± 2.19
				GnRH-A		248	32.06 ± 4.15	3.90 ± 2.23
				PPOS	PCOS	107	29.59 ± 3.40	9.68 ± 4.57
				GnRH-A		178	29.99 ± 3.89	10.03 ± 4.65
				PPOS	POR	52	35.63 ± 4.05	0.86 ± 0.37
				GnRH-A		30	35.50 ± 4.27	0.79 ± 0.44
Pai, 2023 (17)	Retrospective cohort study	2018-2021	PGT-A 27–45 years old	PPOS (DYG)	<38	15	33.4 ± 2.2	3.056 ± 2.206
				GnRH-A	<38	47	33.8 ± 2.9	2.905 ± 2.351
				PPOS (DYG)	>38	19	41.1 ± 1.9	2.166 ± 1.420
				GnRH-A	>38	47	40.9 ± 2.0	2.299 ± 1.871
Çakar,2024 (13)	Retrospective cohort study			PPOS (micronized progesterone)		140 c		
					<35			
					35-37			
					38-40			
					>40			
				GnRH-A		243 c		
					<35			
					35-37			
					38-40			
					>40			
Vidal, 2023/2024 (23, 24)	Prospective cohort study	2019-2022	PGT-A (same population)	PPOS (micronized progesterone)		44		
				GnRH-A		44		
Wan, 2024 (28)	Retrospective cohort study		PGT-A	PPOS	<35	(131,137 c)		
					≥35	(72, 80 c)		
				GnRH-A	<35	(149, 152 c)		
					≥35	(66, 71 c)		
Vaiarelli, 2024 (30)	Retrospective case control study		PGT-A for AMA (matched)	PPOS (NETA)		89		
				GnRH-A		178		
Pittana, 2024 (21)	Retrospective case control study	2021-2022	PGT-A, AMA, DuoStim	PPOS				
				GnRH-A				
Zhou, 2024 (27)	Retrospective cohort study	2020-2023	PGT-A, good prognosis	PPOS (MPA)		250		
				GnRH-A		248		
				GnRH-a		478		
Welp, 2024 (14)	Retrospective cohort study			PPOS		418	35.6 ± 4.6	
				GnRH-A		419	35.7 ± 4.8	
Wang, 2024 (15)	Retrospective case control study	2018-2021		PPOS		320 c	34(31,37)	3.04 (1.79, 4.54)
				GnRH-A		111 c	34(30,39)	3.55 (1.85,5.02)
Yamakami, 2020 (32)	Retrospective case control study	2018-2019		PPOS FP		38, 27 c		
				PPOS LP		49, 26 c		
				GnRH		53,29 c		
Tomioka,2024 (33)	Retrospective case control study	2021-2023	PGT-A	PPOS FP	373			
				PPOS LP	178			

c, cycle.

**Table 2:** The clinical outcomes of the included population.

**Reference**	**Groups**	**Subgroups**	**ER**		**CPR**		**MR**	**LBR**	**PPOS vs other**
Yang, 2020/2022 (18, 19)	PPOS (DYG)		34.9% ± 2.4%						Similar
	GnRH-A		40.2% ± 3.97%						
La Marca, 2020	PPOS (MPA)		21%						Similar
(20)	GnRH-A		21%						
	GnRH-A								
Li, 2021 (25)	PPOS		57.60%					45.50%	Inferior
	GnRH-A		76%					58.30%	
	GnRH-a		67.30%					72.20%	
Abshekenova, 2022 (22)	PPOS		51%		50.70%				Similar
	GnRH-A		59%		40.50%				
Giles, 2022 (10)	PPOS				64.10%		4.70%		Superior
	GnRH-A				62.10%		8.20%		
Giles, 2023 (11)	PPOS		57.90%		(OPR) 50.4%		10.40%		Superior
	GnRH-A		56.40%		(OPR) 47.10%		14.80%		
Huang, 2023 (16)	PPOS (DYG)		33.10%						Inferior
	GnRH-A		48.40%						
	GnRH-a		49.10%						
Rania, 2023 (29)	PPOS		15.4% ± 19.8%				5%	40.40%	Similar
	GnRH-A		14.8% ± 18.2%				8.30%	40.70%	
Wang, 2023 (12)	PPOS		46.54%		(OPR) 46.55%			43.10%	Similar
	GnRH-a		46.04%		(OPR) 52.27%			45.45%	
	GnRH-A		45.78%		(OPR) 50%			39.23%	
Zhou, 2023 (26)	PPOS (MPA)	NOR	N:1.76 ± 1.79					Cu:28.40%	Inferior
	GnRH-A		N:1.97 ± 1.81					Cu:40.70%	
	PPOS	PCOS	N:2.66 ± 2.23					Cu:37.40%	
	GnRH-A		N:2.77 ± 2.28					Cu:46.10%	
	PPOS	POR	N:0.75 ± 1.19					Cu:19.20%	
	GnRH-A		N:0.83 ± 1.02					Cu:16.70%	
Pai, 2023 (17)	PPOS (DYG)	<38	30.1%		50%		10%	40%	Inferior
	GnRH-A	<38	38.5%		55.2%		13.8%	41.4%	
	PPOS (DYG)	>38	5.4%		50%		30%	20%	
	GnRH-A	>38	26.7%		41.9%		12.9%	29%	
Çakar,2024 (13)	PPOS								Similar
		<35	31.10%						
		35-37	29.40%						
		38-40	25.20%						
		>40	14.50%						
	GnRH-A								
		<35	38.80%						
		35-37	30.20%						
		38-40	27%						
		>40	13.80%						
Vidal, 2023/2024 (23, 24)	PPOS (NETA)			13.9% ± 19.3%			24.70%		Similar
	GnRH-A			13.3% ± 17.9%			21.90%		
Wan, 2024 (28)	PPOS	<35		13.80%					Superior
		≥35		54.94%					
	GnRH-A	<35							
		≥35		40.88%					
Vaiarelli, 2024 (30)	PPOS (NETA)							Cu: 24.7%	Similar
	GnRH-A							Cu:21.9%	
Pittana, 2024 (21)	PPOS			24.10%					Similar
	GnRH-A			25.40%					
Zhou, 2024 (27)	PPOS (MPA)							28.40%	Inferior
	GnRH-A							40.70%	
	GnRH-a							42.70%	
Welp, 2024 (14)	PPOS			N:2.4 ± 2.6		70.4%			Superior
	GnRH-A			N:2.2 ± 2.4		64.20%			
Wang, 2024 (15)	PPOS			58.0%		IR: 56.3%	10.0%	52.2%	Similar
	GnRH-A			52.4%		IR: 57.8%	7.8%	58.0%	
Yamakami, 2020 (32)	PPOS FP								Similar
	PPOS LP								
	GnRH-A								
Tomioka,2024 (33)	PPOS FP			37.10% ± 0.015					Similar
	PPOS LP			32% ± 0.02					

N, number of euploid blastocyst; Cu, cumulative.

Aneuploidy rate = number of aneuploid embryos/total number of tested embryos.

## 3 Results

### 3.1 In the general population

#### 3.1.1 In the unclassified population

In 2022, Giles conducted a multicenter observational study encompassing 4,961 non-oncological fertility preservation cycles and 12,461 preimplantation genetic testing for aneuploidy (PGT-A) cycles. Notably, despite a reduced number of biopsied embryos in the PPOS group, the study revealed comparable aneuploidy rates, implantation rates (IR), and clinical pregnancy rates (CPR) to those of the gonadotropin-releasing hormone antagonist (GnRH-A) group. Moreover, the PPOS group exhibited significantly lower miscarriage rates (MR) (10). A subsequent multi-center retrospective observational cohort study by Giles analyzed 1652 social fertility preservation cycles and 5661 PGT-A cycles. The PPOS group had comparable metaphase II (MII) oocytes, biopsied embryos, EER, or ongoing pregnancy rate (OPR) and a lower clinical miscarriage rate compared to the GnRH-A group (11). Wang’s retrospective cohort study also had similar conclusions. The study included 608 PGT-A cycles, with 146 women in the PPOS group, 160 in the GnRH agonist (GnRH-a) group, 302 in the GnRH-A group, and 267 corresponding first frozen embryo transfer (FET) cycles were analyzed. The results demonstrated comparable EER per MII oocyte, with no significant differences in reproductive outcomes, including live birth rate (LBR) among the three groups (12). Recently, Çakar conducted a multicenter retrospective cohort study, examining 1,425 PGT-A-tested blastocysts derived from 383 cycles. The study revealed that there were no significant differences in EER per blastocyst tested or HCG positivity rates per transferred embryo between the PPOS and GnRH-A groups (13). In another cohort trial, Welp’s investigation demonstrated the efficacy of medroxyprogesterone acetate (MPA) in effectively inhibiting ovulation, while achieving comparable cycle outcomes and reproductive success rates. Furthermore, MPA conferred several patient-centered benefits, including cost savings, a reduction in the number of monitoring visits, and a decrease in the frequency of injections (14). More recently, Wang’s study, which encompassed a substantial sample size of 431 PGT-A cycles, provided additional insights into this area. Specifically, the study found no statistically significant differences in aneuploidy embryo rates, clinical pregnancy rates, or cumulative LBR between the PPOS protocol (n=320) and the GnRH-A protocol (n=111) (15).

Despite these favorable outcomes, differing perspectives existed. Huang observed that, although baseline characteristics were comparable, the average euploid blastocyst count and EER were lower in the PPOS group compared to the GnRH-a and GnRH-A groups in 457 PGT-A cycles. However, it is crucial to note that patients in the PPOS group had a significantly higher mean age than those in the other two groups (16). Similarly, a retrospective cohort study by Pai analyzed 128 PGT-A cycles at a university hospital-affiliated fertility center between 2018 and 2021. Among elderly patients (≥38 years old), the PPOS group showed lower blastocyst formation and euploidy rates compared to the GnRH-A group. However, the sample size was small (only 34 cases in the PPOS group), and the study was retrospective, necessitating cautious interpretation of the results (17). Collectively, the PPOS protocol yields reproductive outcomes non-inferior to those of GnRH-a and GnRH-A protocols in the unclassified population.

#### 3.1.2 In the matched or self-controlled population

To address potential biases, several studies employed matched population. Yang conducted a single-center retrospective cohort study comparing 390 PGT-A cycles with oral dydrogesterone (DYG) to 390 cycles with the GnRH-A protocol among 780 initial PGT-A cycles. After propensity matching for age, body mass index (BMI), and Anti - Müllerian Hormone (AMH), the study found similar EER per biopsy between the two groups (18, 19). A prospective non-inferiority, age-matched case-control study by La Marca, involving 192 patients and 785 blastocysts from 1867 injected oocytes, demonstrated that the PPOS protocol achieved comparable euploid blastocyst formation rates per oocyte compared to GnRH-A protocol. The study also found similar percentages of patients with euploid embryos and total euploid blastocysts per patient, further supporting the effectiveness of the PPOS protocol (20). A recent retrospective study by Pittana compared 138 patients undergoing PPOS-DuoStim with 138 matched patients undergoing conventional-DuoStim protocol. The study found comparable EER per biopsied blastocyst between the PPOS-DuoStim group (24.1%) and the conventional-DuoStim group (25.4%) (21). In self-controlled population, the following studies had similar conclusions. In a retrospective cohort study by Abshekenova, which compared 201 IVF/ICSI cycles using self-matched donor oocytes, no significant differences were observed between the consecutive PPOS and GnRH-A groups in gonadotropin dosage, oocyte maturity, fertilization rates, blastocyst formation rates, blastocyst with top quality, EER, or pregnancy rates (22). Lastly, a prospective study by Vidal involving 44 women who underwent two consecutive ovarian stimulation protocols—GnRH-A and PPOS with oral micronized progesterone—within a six-month period revealed comparable euploid means between the two groups. Although there was a slight difference in EER per biopsied embryo (29% in PPOS *vs* 35% in GnRH-A), the PPOS group had more oocytes, MIIs, and 2 pronucleus (PNs) (23, 24). In summary, the PPOS protocol can achieve reproductive outcomes comparable to, if not superior to, those of the GnRH-a and GnRH-A protocol.

### 3.2 In specific population

#### 3.2.1 In population with a good prognosis

Li observed a notable decline in both the EER (57.6% *vs* 76.0% *vs* 67.3%) and LBR (45.5% *vs* 58.3% *vs* 72.2%) among young patients (<35 years old) utilizing PPOS protocol, in comparison to those using GnRH-A and GnRH-a, respectively (25). Another retrospective cohort study compared the cumulative LBR of PPOS and GnRH-A protocols in PGT cycles among 865 patients from three population (normal ovarian response (NOR), polycystic ovary syndrome (PCOS), poor ovarian response (POR)). Results showed that in NOR and seemingly in PCOS, PPOS had lower or seemingly lower cumulative LBR compared to GnRH-A, while in POR they were comparable (26). In the retrospective cohort study conducted by Zhou, comparing PPOS with GnRH analogues in patients with a good prognosis undergoing PGT cycles, the PPOS protocol was found to be adversely associated with both cumulative LBR and blastocyst quality. While perinatal outcomes were comparable across PPOS, GnRH-A, and GnRH-a groups, the time to live birth was longer with PPOS protocol compared to GnRH-A protocol (27). In summary, the PPOS protocol may be less effective than conventional COH approaches.

#### 3.2.2 In population with a poor prognosis

In patients with a poor prognosis, the potential advantages of PPOS have gained significant attention. A recent study by Wan revealed that higher EER and a decreased mosaicism rate when compared to GnRH-A protocol within the older patient cohort. This disparity suggests that exogenous progesterone administration in the PPOS protocol may exert a positive influence on oocyte meiosis or early embryo mitosis in older patients. Intriguingly, among younger patients, no statistically significant differences were observed between PPOS and GnRH-A treatments, and no correlation was established between the ovarian stimulation protocol and ongoing pregnancy rates (28). Adding to this body of evidence, a matching case-control study conducted by Rania focused on advanced-maternal-age (AMA) women undergoing PGT-A. Across key performance indicators such as fertilization rates, blastulation rates, EER, and both MR and LBR, similar outcomes were reported for both PPOS and GnRH-A groups. Furthermore, cumulative LBR were also comparable between the two protocols (29). Vaiarelli et al. conducted a retrospective matched case-control study comparing oocyte competence between norethisterone acetate-primed ovarian stimulation and the conventional GnRH antagonist protocol in advanced maternal age (AMA) women undergoing preimplantation genetic testing for aneuploidy (PGT-A). Results revealed comparable EBR per MII oocytes, blastocyst morphology, developmental rates, clinical outcomes, and cumulative LBR between the PPOS and control groups (30). Expanding the scope of investigation, Vaiarelli’s another retrospective study compared PPOS-DuoStim with antagonist-DuoStim cycles in 444 couples undergoing PGT-A at a private center, with a mean age of 40. This study demonstrated comparable embryological and clinical outcomes, including similar euploid blastocyst rates per MII oocyte and cumulative LBR between the two protocols (31). It could be found that among those with poor prognoses, the PPOS protocol has shown comparable, even superior, euploid embryo rates and reproductive outcomes.

### 3.3 The timing of cycle initiation

Does the initiation of PPOS in the follicular phase versus the luteal phase matter? In 2020, Yamakami et al. conducted a retrospective study comparing different pituitary suppression regimens during ovarian stimulation. The results revealed that the administration of progestins, irrespective of whether initiated in the follicular phase (FP) or luteal phase (LP), did not exert any significant influence on chromosomal rearrangements, pregnancy outcomes, or endometrial priming. Notably, compared to GnRH-A (72.9%), both FP and LP progestin groups exhibited a substantial increase in fertilization rates (FP: 86.0% *vs* LP: 90.5%) (32). In 2024, Tomioka et al. performed a retrospective observational study encompassing 551 PGT-A cycles. This study found no remarkable differences in oocyte competence and embryonic ploidy between FP and LP cycles among patients undergoing PPOS, regardless of their ovarian response. Specifically, the rates of MII oocytes, fertilization, high-quality blastocysts, and EER were comparable between the two groups (33). It appears that the initiation of PPOS in the follicular phase versus the luteal phase does not affect reproductive outcomes.

## 4 Discussion

Our review may emerge as a pioneering endeavor, comprehensively evaluating the impact of PPOS protocol on EER and reproductive outcomes in PGT cycles. By conducting subgroup analyses, we have gained deeper insights into the performance of PPOS. Furthermore, this review underscores that the initiation of the PPOS protocol does not significantly impact EER or reproductive outcomes. This may broaden the potential applications of PPOS, particularly in patients requiring fertility preservation measures.

In the general population, the PPOS protocol has exhibited comparable reproductive outcomes to those attained through alternative COH protocols. This conclusion is further reinforced by findings from matched and self-controlled population. This is consistent with the conclusions drawn in non-PGT cycles. A recent meta-analysis emphasizes the comparable LBR or OPR per embryo transfer between cycles utilizing the PPOS protocol and those employing the GnRH-A protocol (34).

When stratifying analyses among patient subgroups, the picture changes. In patients with a good prognosis, several studies reported a potential underperformance of the PPOS protocol compared to traditional COH methods. The underlying mechanism may be attributed to the detrimental effects of elevated progesterone levels on oocyte quality. Studies have demonstrated that progesterone can impair blastocyst formation rates in bovine cumulus-oocyte complexes (35), inhibit meiosis resumption in mouse oocytes, and adversely affect oocyte maturation and quality (36). It was also reported that progesterone could suppress the rate of mitosis and apoptosis on granulosa cells via phosphatidylinositol-3 kinase/protein kinase B and mitogen-activated protein kinase pathway (37, 38). Notably, scRNA-seq analysis revealed significantly higher expression of 12 mitochondrial DNA genes in mural granulosa cells of the PPOS group compared with the GnRH antagonist group, which may mechanistically underlie the lower live birth rates observed in PPOS cycles (39).

Conversely, in patients with a poor prognosis, the PPOS protocol has demonstrated comparable or even superior euploid embryo rates and reproductive outcomes, aligning with findings from studies conducted in non-PGT cycles. A self-controlled study among infertile patients with diminished ovarian reserve (DOR) revealed that MPA effectively suppressed LH surges, leading to enhanced outcomes, including LBR (40). Furthermore, a meta-analysis encompassing 14 studies with 4,182 participants found statistically significant improvements in clinical pregnancy rate, optimal embryo rate, and cumulative pregnancy rate when PPOS was compared to clomiphene citrate/letrozole plus gonadotropin protocol for women with DOR (5). The reasons behind these benefits are multifaceted. Patients with a poor prognosis are prone to premature or occult LH surges, which adversely affect follicle quality. The PPOS protocol effectively mitigates these adverse effects by suppressing LH surges from the outset. This is supported by a study demonstrating that MPA significantly suppressed LH surges and improved reproductive outcomes compared to the clomiphene citrate protocol (40). Furtherly, a retrospective analysis of 102 patients in POSEIDON group 4 found lower LH levels on the trigger day and a higher proportion of MII oocytes in the PPOS group compared to the minimal stimulation group (41). At the molecular level, progesterone may improve the follicular microenvironment by regulating the expression of specific microRNAs, such as miR-4261 and miR-6869-5p, in granulosa cells (42). MPA could enhance ovulation rates by upregulating the messenger RNA expression of gap junction protein alpha 1 and vascular endothelial growth factor in follicles (43). Additionally, DYG has been shown to stimulate oocyte maturation and ovulation by increasing the concentrations of acylcarnitines, lysophospholipids, urea, putrescine, and free amino acids in the ovary, via purinergic signaling and arachidonic acid metabolism pathways (44).

From the aforementioned, it is evident that progesterone exerts both beneficial and detrimental effects on oocyte and embryo quality at the molecular level, with its primary role likely influenced by the surrounding microenvironment. This may explain why, in the general population, which likely includes a mix of patients with varying reproductive characteristics, the PPOS protocol demonstrates no significant difference in EER and reproductive outcomes compared to conventional COH protocols.

Nevertheless, it is critically important to recognize the divergent conclusions presented in numerous studies. Such discrepancies are likely attributable to variations in study designs, participant age, ovarian function, sample sizes, as well as differences in the formulations and dosages of progesterone used. Previous studies have indicated that within controlled ovarian hyperstimulation (COH) regimens, different doses and types of progesterone result in varying pregnancy outcomes (34, 45). Additionally, several key clinical variables—including causes of infertility, duration of infertility, male semen quality, and PGT testing methods—were not consistently reported across the studies. Therefore, these results should be interpreted with caution.

## 5 Conclusion

In conclusion, while PPOS has demonstrated comparable EBR and reproductive outcomes to conventional COH protocols in general population, its application in population with a poor prognosis yields comparable or even better reproductive outcomes, whereas cautious use is warranted in those with a good prognosis. The timing of PPOS cycle initiation, had no significant impact on clinical outcomes in patients with diverse ovarian responses. It is imperative to make individualized treatment plans based on patients’ characteristics and needs.

## Data Availability

The original contributions presented in the study are included in the article/supplementary material. Further inquiries can be directed to the corresponding authors.
